# Data sharing and management in 21st century cancer research

**DOI:** 10.1002/1878-0261.13385

**Published:** 2023-02-13

**Authors:** Kevin M. Ryan

**Affiliations:** ^1^ Molecular Oncology Editorial Office FEBS Press Cambridge UK; ^2^ Cancer Research UK Beatson Institute Glasgow UK; ^3^ School of Cancer Sciences University of Glasgow Glasgow UK

A central facet of scientific endeavour is that we must share our discoveries. Even in the ‘Ivory Tower’ science of previous centuries where scientists would often work alone, they would still disseminate their findings by attending events at learned societies and by publishing their work in scientific journals; otherwise, one could argue, if the discovery was not recorded, how do we know it happened? To this day, things are much the same, but also very different. We still need to meet and discuss our science at conferences and other events, so that we can share our ideas and early data to facilitate the progress of discovery. This also enables us to communicate with those whom we do not on a daily basis, e.g. through events that bring together basic scientists with those who undertake more translational research or are involved in clinical trials. In addition, we still need to report our findings in peer‐reviewed journals. The pathways to share data and information are though more varied with people choosing to share their findings via social media platforms, webinars and other forms of digital media. The digital era has also changed the way we can ask scientific questions, with the ability to generate and analyse very large sets of data that have enormous power to make discoveries that were previously not possible. While the conclusions from these new large data studies can be easily communicated, the ability to manage and share the data, and more so, the metadata behind these studies are currently a burgeoning problem for many areas of research, including cancer research. This is not only a technical problem in handling the huge amounts of data involved, but in some cases also a legal problem when factors such as General Data Protection Regulations (GDPR, https://gdpr.eu/) need to be considered.

To address these issues, we need discussions between leaders in the field, as well as the general cancer research community, to bring the key points to light and to see whether solutions can be found. To this end, *Molecular Oncology* has partnered with the European Academy of Cancer Sciences (EACS, https://www.europeancanceracademy.eu/) and the European Association for Cancer Research (EACR, https://eacr.org/) to host a keynote session and discussion forum on ‘The Challenges of Data Management and Sharing in Translational Cancer Research’ at this year's EACR Congress. The EACR Congress is a pre‐eminent event in European basic and translational cancer research, and this year's Congress will take place in Torino, Italy; 12–15 June 2023 (https://www.eacr2023.org/). The session on data management and sharing will involve three keynote speakers, which will be followed by a question‐and‐answer session open to all attending delegates. I will also be present at the Congress to take part in a ‘Meet the Editor’ session, as will other members of the *Molecular Oncology* team. So, please come and join us at EACR 2023 to hear and contribute to the debate on 21st century data management and sharing and to discuss with the *Molecular Oncology* team your latest discoveries, which you may consider submitting to our nonprofit journal.

## Conflict of interest

The author declares no conflict of interest.

